# A Horrible Labor

**Published:** 1876-02

**Authors:** 


A Horrible Labor.—The *Allgemeine Medicinische Central-Zeitung* quotes the following remarkable case from Warsaw. At the railway station of Woloschin, the duties of signaller were performed by a woman, who was in an advanced stage of pregnancy. About five o'clock on the morning of November 24th, a train was approaching, and the woman went on the line to make the signals. She was probably seized with labor-pains as the train approached; for a few hours later some laborers found her body literally cut in two, the upper part being completely smashed, while in the sand at the side of the rails lay a child, living and crying, but quite uninjured. At the examination it was decided that the birth of the child must have taken place after the death of the mother, or while she was dying.

